# Molecular docking with Gaussian Boson Sampling

**DOI:** 10.1126/sciadv.aax1950

**Published:** 2020-06-05

**Authors:** Leonardo Banchi, Mark Fingerhuth, Tomas Babej, Christopher Ing, Juan Miguel Arrazola

**Affiliations:** 1 Xanadu, 372 Richmond St W, Toronto, ON M5V 1X6, Canada.; 2 ProteinQure Inc., 192 Spadina Ave, Toronto, ON M5T 2C2, Canada.

## Abstract

Gaussian Boson Samplers are photonic quantum devices with the potential to perform intractable tasks for classical systems. As with other near-term quantum technologies, an outstanding challenge is to identify specific problems of practical interest where these devices can prove useful. Here, we show that Gaussian Boson Samplers can be used to predict molecular docking configurations, a central problem for pharmaceutical drug design. We develop an approach where the problem is reduced to finding the maximum weighted clique in a graph, and show that Gaussian Boson Samplers can be programmed to sample large-weight cliques, i.e., stable docking configurations, with high probability, even with photon losses. We also describe how outputs from the device can be used to enhance the performance of classical algorithms. To benchmark our approach, we predict the binding mode of a ligand to the tumor necrosis factor-α converting enzyme, a target linked to immune system diseases and cancer.

## Teaser

Photonic quantum devices called Gaussian Boson Samplers can be programmed to predict molecular docking configurations.

## INTRODUCTION

In his lecture “Simulating Physics with Computers” (*1*), Richard Feynman famously argued that classical computing techniques alone are insufficient to simulate quantum physics. Since then, substantial progress has been made in formalizing this intuition by finding explicit examples of quantum systems whose classical simulation can be convincingly shown to require exponential resources. An example is Boson Sampling, first introduced by Aaronson and Arkhipov (*2*). In this paradigm, identical photons interfere by passing through a network of beam splitters and phase shifters and are subsequently detected at the output ports of the network. Despite the simplicity of this model, it has been shown that, under standard complexity theoretic conjectures, generating samples from the output photon distribution requires exponential time on a classical computer (*2*–*4*). Several variants of Boson Sampling have been proposed that aim at decreasing the technical challenges with its experimental implementation (*5*–*8*).

Most efforts in the study of Boson Sampling have been focused on its viability to disprove the Extended Church-Turing thesis (*9*), not on its potential practical applications. Nevertheless, it is possible to ask: If Boson Sampling devices are powerful enough that they cannot be simulated with conventional computers, is there a way of programming them to perform a useful task? Practical applications of Boson Sampling have already been reported. In (*10*), it was shown that a Boson Sampling device can be used to efficiently estimate the vibronic spectra of molecules, a problem for which, in general, no efficient algorithm is known. Proof-of-principle demonstrations have also been reported (*11*, *12*). In addition, (*13*–*15*) discuss how a specific model known as Gaussian Boson Sampling (GBS) can be used in combinatorial optimization problems concerned with identifying large clusters of data.

Molecular docking is a computational method for predicting the optimal interaction of two molecules, typically a small-molecule ligand and a target receptor. This method works by searching the configurational space of the two molecules and scoring each pose using a potential energy function. Using molecular structures to determine stable ligand-receptor complexes is a central problem in pharmaceutical drug design (*16*, *17*). Several techniques for finding stable ligand-receptor configurations have been developed, including shape-complementarity methods (*18*–*21*) and molecular simulation of the ligand-receptor interactions (*22*), which vary in their computational requirements. For high-throughput virtual screening of large chemical libraries, it is desirable to search and score ligand-receptor configurations using as few computational resources as possible (*23*). However, it is important to keep in mind that accurate docking in the absence of a reliable scoring function is a major challenge. Molecular docking is related to the prediction of molecular similarity for ligand-based virtual screening, which has been formulated as a graph theoretic problem for quantum annealers (*24*, *25*).

In this work, we show that GBS can be used to find docking configurations between ligands and receptors. We extend the binding interaction graph approach, where the problem of identifying docking configurations can be reduced to finding large clusters in weighted graphs (*26*, *27*). We then show how GBS devices can be programmed to sample from distributions that assign large probabilities to these clusters, thus helping in their identification. The best docking configurations are selected based on the weight of the corresponding clique. GBS devices therefore enhance the search for stable configurations—which correspond to cliques—while the scoring function is implicit in the weights of the input graph. Docking configurations can be obtained by direct sampling or by hybrid algorithms where the GBS outputs are post-processed using classical techniques. The goal of this paper is to demonstrate that GBS devices can improve the sampling of docking configurations, whereas it does not address the difficulties associated with scoring functions (*28*, *29*), which represent the major hurdle in accurate docking. We apply our method to find molecular docking configurations for a known ligand-receptor interaction (*30*), using a simplified representation to make the problem solvable through numerical simulations. Several therapeutic agents targeting this protein have entered into clinical trials for both cancer and inflammatory diseases (*31*). A different protein structure is studied in the Supplementary Materials.

## RESULTS

Before presenting our results, we provide relevant background information on molecular docking. Readers not familiar with GBS may read Materials and Methods.

### Molecular docking

Molecular docking is a computational tool for rational structure-based drug discovery. Docking algorithms predict noncovalent interactions between a drug molecule (ligand) and a target macromolecule (receptor) starting from unbound three-dimensional structures of both components. The output of such algorithms is predicted three-dimensional orientations of the ligand with respect to the receptor binding site and the respective score for each orientation. Reliable determination of the most probable ligand orientation, and its ranking within a series of compounds, requires accurate scoring functions and efficient search algorithms (*28*). The scoring function contains a collection of physical or empirical parameters that are sufficient to score binding orientation and interactions in agreement with experimentally determined data on active and inactive ligands. The search algorithm describes an optimization approach that can be used to obtain the minimum of a scoring function, typically by scanning across translational and rotational degrees of freedom of the ligand in the chemical environment of the receptor. In the simplest case, both the ligand and the receptor can be approximated as rigid bodies, but more accurate methods can account for inherent flexibility of the ligand and receptor (*21*). As is the case for most molecular modeling approaches, a trade-off exists between accuracy and speed.

High-performance algorithms enable molecular docking to be used for screening large compound libraries against one or more protein targets. Molecular docking and structure-based virtual screening are routinely used in pharmaceutical research and development (*32*). However, evaluating billions of compounds requires accurate and computationally efficient algorithms for binding pose prediction. Widely used approaches for molecular docking use heuristic search methods [simulated annealing (*33*) and evolutionary algorithms (*34*)] and deterministic methods (*35*). In one combinatorial formulation of the binding problem used in the DOCK 4.0, FLOG, and SQ algorithms, an isomorphous subgraph matching method is used to generate ligand orientations in the binding site (*26*, *36*–*38*). In this formulation of the binding problem, both the ligand and the binding site of the receptor are represented as complete graphs. The vertices of these graphs are points that define molecular geometry, and edges capture the Euclidean distance between these points. To strike a balance between the expressiveness of the graph and its size, we reduce the all-atom molecular models of the ligand and receptor to a pharmacophore representation (*39*).

**Fig. 1:**
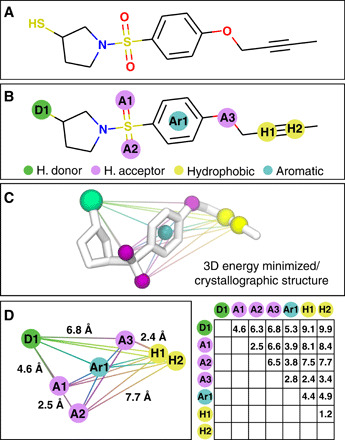
Construction of the labeled distance graph for a ligand molecule. (**A**) Planar structure of the ligand molecule. Pharmacophore points of the molecule (**B**) are identified, and their pairwise distance is measured using the known three-dimensional (3D) structure (**C**). This information is combined in the labeled distance graph for the ligand molecule (**D**), where vertices represent the pharmacophore points and edge weights of their respective pairwise distance [the complete weight matrix is on the right of (D)].

A pharmacophore is a set of points that have a large influence on the molecule’s pharmacological and biological interactions. These points may define a common subset of features, such as charged chemical groups or hydrophobic regions, that may be shared across a larger group of active compounds. For the purposes of this study, we define six different types of pharmacophore points: negative/positive charge, hydrogen-bond donor/acceptor, hydrophobe, and aromatic ring. In the graph representation, the type of the pharmacophore point is preserved as a label associated with its vertex. Hence, we refer to this molecular graph representation as a labeled distance graph (see also section S2). As illustrated in Fig. 1, a labeled distance graph is constructed as follows for both the ligand and receptor:

1) Heuristically identify pharmacophore points likely to be involved in the binding interaction. These form the vertices of the graph.

2) Add an edge between every pair of vertices and set its weight to the Euclidean distance between the pharmacophore points they represent.

3) Assign a label to every vertex according to the respective type of pharmacophore point it represents.

### Mapping molecular docking to maximum weighted clique

The labeled distance graphs described in the previous section capture the geometric three-dimensional shapes and the molecular features of both the protein binding site and the ligand that interacts with it. In this section, akin to (*26*), we combine these two graphs into a single binding interaction graph. Subsequently, we reduce the molecular docking problem to the problem of finding the maximum weighted clique.

If two pharmacophore points are interacting, they form a contact. A binding pose can be defined by a set of three or more contacts that are not colinear. We model contacts as pairs of interacting vertices of the labeled distance graphs of the ligand and the binding site. Consider the labeled distance graph *G_L_* of the ligand and the labeled distance graph *G_B_* of the binding site, with their vertex sets *V_L_* and *V_B_*, respectively. A contact is then represented by a single vertex *c_i_* ∈ *V_L_* × *V_B_*. The set of possible contacts forms the vertices of the binding interaction graph. In principle, any pharmacophore point of the ligand could be interacting with any pharmacophore point of the binding site, and therefore, we have to consider every possible pair of corresponding interacting vertices. Hence, the number of vertices of the binding interaction graph is *nm*, where *n* is the number of vertices of the labeled distance graph *G_L_* and *m* is the number of vertices of the labeled distance graph *G_B_*.

The goal of the binding interaction graph is to model possible binding poses via sets of contacts. However, not every combination of contacts is physically realizable. Two contacts are not being compatible if their mutual realization would violate the geometrical shapes of the ligand and the binding site. To model this, the binding interaction graph contains an edge between two contacts if and only if they are compatible. As a result, a pairwise compatible set of contacts, i.e., such as would arise from a true binding pose, forms a complete subgraph of the binding interaction graph. A complete subgraph, also called a clique, in a graph *G* is a subgraph where all possible pairs of vertices are connected by an edge.

**Fig. 2:**
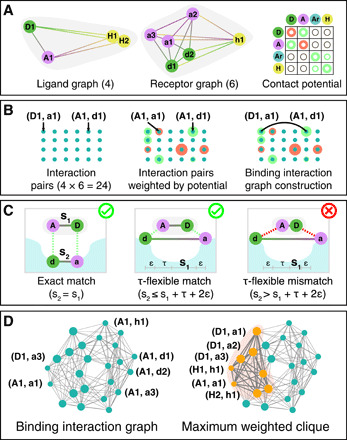
Construction of the binding interaction graph. (**A**) Inputs for the construction of the binding interaction graph—two labeled graphs (one for the ligand and one for the receptor) and corresponding contact potential that captures the interaction strength between different types of vertex labels. We denote vertices on the ligand and receptor with uppercase and lowercase letters, respectively. The binding interaction graph is constructed (**B**) by creating a vertex for each possible contact between ligand and the receptor weighted by the contact potential. Pairs of vertices that represent compatible contacts [see (**C**) for various scenarios] are connected by an edge. The resulting graph is then used to search for potential binding poses (**D**). These are represented as complete subgraphs—also called cliques—of the graph, as they form a set of pairwise compatible contacts. The heaviest vertex-weighted cliques represent the most likely binding poses (maximum vertex-weighted clique depicted in orange).

The compatibility of contacts is captured by the notion of τ flexibility, which is illustrated in Fig. 2 (see also section S2). Although both the ligand and the binding site can exhibit a certain amount of flexibility, in general, geometric distances between two contacts have to be approximately the same on both the ligand and the binding site. Two contacts (*v*_*l*_1__, *v*_*b*_1__) and (*v*_*l*_2__, *v*_*b*_2__) form a τ flexible contact pair if the distance between the pharmacophore points on the ligand (points corresponding to vertices *v*_*l*_1__ and *v*_*l*_2__) and the distance between the pharmacophore points on the binding site (points corresponding to vertices *v*_*b*_1__ and *v*_*b*_2__) do not differ by more than τ + 2є (see Fig. 2C). The constants τ and є describe the flexibility constant and interaction distance, respectively. The role of τ and є is therefore to determine which edges actually appear in the binding interaction graph.

To model varying interaction strengths between different types of pharmacophore points, we associate a different weight to every vertex of the binding interaction graph. The weights are derived using the pharmacophore labels that are captured in the labeled distance graphs of the ligand and the binding site. Given a set of labels –, a potential function κ:L×L→ℝ is applied to compute the weights of the individual vertices. This allows us to bias the algorithm toward stronger intermolecular interactions. Potential functions can be derived in several ways, ranging from pure data-based approaches such as statistical or knowledge-based potentials (*40*–*42*) to quantum-mechanical potentials (*16*). Details of the potential used in this study are described in “Numerical results.”

Hence, under the model derived in this study, the most likely binding poses correspond to vertex-heaviest cliques in the binding interaction graph. The problem of finding a maximum weighted clique is a generalization of the maximum clique problem of finding the clique with the maximum number of vertices. When *G* has *n* vertices, the number of possible subgraphs is $O(2^{n})$, so a brute force approach becomes rapidly infeasible for growing values of *n*. The max clique decision problem is NP-hard (*43*): As such, unless *P* = NP, in the worst case, any exact algorithm runs for superpolynomial time before finding the solution. There are deterministic and stochastic classical algorithms for finding both the maximum cliques and maximum weighted cliques or for finding good approximations when *n* is large (*44*).

It is crucial to understand that the outputs of this method are only as good as the quality of the binding interaction graph as a model for the docking problem. Graphs built exclusively from subsets of pharmacophores that are not involved in the correct binding pose will lead to incorrect pose predictions. Conversely, by construction, the largest weighted clique in accurate graphs will correspond to the correct binding pose. It is a feature of our approach that different values of the parameters τ and є result in the same binding interaction graph and hence the correct maximum weighted clique, as shown in table S2. Following our procedure, the predicted binding configuration can be scored and ranked among a set of alternate molecules, or a continuous-space representation of the complex can be derived using distance restraints derived from the solution using an industry-standard docking algorithm. Our capability to couple conformational search and scoring through parameterization by weights of the graph suggests that we may not be subject to bottlenecks arising from scoring function evaluation. We see our approach as synergistic with parallelized continuous space methods, such as restrained docking or alternate hybrid protocols.

### Max weighted clique from GBS

**Fig. 3:**
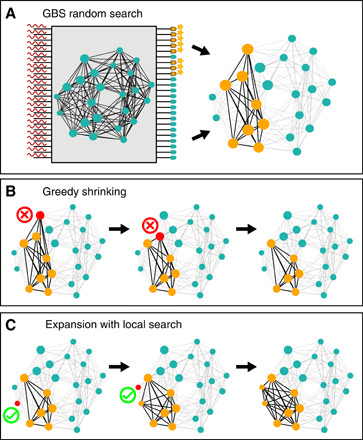
Schematics of the protocol. Squeezed light is injected from the left into a GBS device, which is programmed to sample from a vertex-weighted graph. The presence (star) or absence of photons is measured by detectors on the right. GBS random search (**A**): On the basis of the ports where photons have been detected, we construct a subgraph (yellow vertices and dark edges) and check if it is a clique. If it is not a clique, greedy shrinking (**B**) iteratively removes a vertex (red node with a cross) until the remaining ones form a clique. Two shrinking iterations are shown in (B) from left to right. In local search (**C**), the found clique is expanded by iteratively adding, as long as possible, a neighboring vertex (red node with a tick) to get a bigger clique.

In this section, we show that a GBS device can be programmed to sample from a distribution that outputs the max weighted clique with high probability. The main technical challenge is to program a GBS device to sample, with high probability, subgraphs with a large total weight that are as close as possible to a clique. As shown in Materials and Methods and depicted in Fig. 3, a GBS device is composed of two main parts. In the first part, a chosen quantum Gaussian state is generated via squeezing, phase shifters, and beam splitters. The chosen state is identified by an *M* × *M* matrix that is related to the covariance matrix of the Gaussian state, where *M* is the number of optical modes. The second part is made by photon-counting detectors that measure the number of photons coming out of each mode. To find a suitable input matrix for GBS, consider a graph with *M* vertices and with graph Laplacian *L* = *D* − *A*, where *D* is the degree matrix and *A* is the adjacency matrix. The normalized Laplacian (*45*) $\tilde{L}=D^{-1/2}LD^{-1/2}$ is positive semidefinite, and its spectrum is contained in [0, 2]. More generally, we define a rescaled matrix

B=Ω(D−A)Ω (1)

where Ω is a suitable diagonal matrix. If the largest entry of Ω is bounded as shown in section S1, then the spectrum of *B* is contained in [0, *c*], where *c* ≤ 1 can be tuned depending on the maximum amount of squeezing obtainable experimentally. Using the decoupling theorem from section S1, we find that a GBS device can be programmed to sample from the distribution

$$p(S)\propto {\left[\text{det}(\Omega_{S})\text{Haf}(A_{S})\right]}^{2}$$ (2)

where Haf refers to the matrix Hafnian and we consider outputs *S* = (*n*_1_, …, *n_M_*) with *n_j_* detected photons in mode *j*, so *N* = ∑*_j_n_j_* is the total number of photons. When we focus on the collision-free subspace, where *n_j_* ≤ 1, the dependence on the diagonal matrix *D* disappears so we may focus on programming GBS with a rescaled adjacency matrix Ω*A*Ω. As shown in Fig. 3, from a GBS sample *S*, we construct the subgraph *H* of *G* made by vertices *j* with *n_j_* = 1. The matrix *A_S_* is the *N* × *N* adjacency matrix of *H*. The Hafnian of an adjacency matrix is maximum for the complete graph, namely, when *H* is a clique. Therefore, for a fixed total number of photons *N*, the Hafnian term maximizes the probability of detecting photon configurations that correspond to a clique.

Different choices are possible for the weighting matrix Ω. For an unweighted graph, convenient choices are either a constant Ω or Ω ∝ *D*. In the former case, det Ω*_S_* = *c^N^* for *c* < 1, so the parameter *c* can be tuned by squeezing to penalize larger *N*, i.e., larger subgraphs (see section S1.3). In the latter case, det Ω = *c^N^* det *D* is proportional to the Narumi-Katayama index (*46*), which describes some topological properties of the graph. Similarly to the Hafnian, it is maximum when *H* is a clique.

For a vertex-weighted graph, we can use the freedom of choosing Ω to favor subgraphs with larger total weight. There are multiple ways of introducing the weights *w_j_* in Ω and a convenient choice is

$$\Omega_{\mathrm{ii}}=c(1+\alpha w_{i})$$ (3)

where *c* is a normalization to ensure the correct spectral properties and α > 0 is a constant. When α is small, the determinant term det Ω*_S_* ≈ 1 + α∑_*j*:*n_j_* = 1_*w_j_* is large when the subgraph *H* has a large total weight. This is useful for the max weighted clique problem as it introduces a useful bias in the GBS probability of Eq. 2 that favors heavier subgraphs. However, if α is too large, the Hafnian term in Eq. 2 becomes less important and GBS will sample heavy subgraphs that typically do not contain cliques. To prevent this occurrence, the parameter α must be chosen carefully. Ideally, the weights should give a positive bias to heavy cliques but should not favor heavy subgraphs that are not cliques. More details are discussed in section S1.

### Hybrid algorithms

GBS devices can, in principle, have a very high sampling rate—primarily limited by detector dead time—so just by observing the photon distribution, it is possible to extract the maximum weighted clique for small enough graphs. We call this simple strategy GBS random search—see Fig. 3 for a graphical explanation of the method. However, selecting photon outcomes that correspond only to cliques means wasting samples that are potentially close to the solution. An optimally programmed GBS device will sample from both the correct solution and neighboring configurations with high probability. Therefore, we propose two algorithms to post-process all GBS data that incur an overhead in run time but are especially useful for finding cliques in larger graphs.

#### Greedy shrinking

Starting from an output subgraph *H* from GBS, vertices are removed based on a local rule until a clique is found—see Fig. 3 for a graphical explanation of the method. Removal is based on vertex degree and weight. Vertices with small degree are unlikely to be part of a clique, making them good candidates to be discarded. The role of the weights is less straightforward: Vertices with low weight may not be part of the max weighted clique, but this assumption may be incorrect if the clique is made by a heavy core together with a few light vertices. Because of this, vertex degree is prioritized over vertex weight during the greedy shrinking stage. More precisely, the algorithm proceeds as follows:

1) From a GBS outcome, build a subgraph *H* with vertices corresponding to the detectors that “click.”

2) If *H* is a clique, return *H*.

3) Otherwise, set *v* as the set of vertices in *H* with smallest degree.

4) Set *w* as the subset of *v* with lowest weight.

5) Remove a random element of *w* from *H* and go back to step 2.

#### Expansion with local search

GBS provides high-rate samples from max cliques, and greedy shrinking enhances the probability of finding a solution via classical post-processing of sampled configurations. We may increase the probability of finding the solution even further at the cost of a few more classical steps. This is done by using a local search algorithm that tries to expand the clique with neighboring vertices, as shown also in Fig. 3. We use algorithms such as dynamic local search (DLS) (*47*) and phased local search (PLS) (*48*) that are among the best-performing classical algorithms for max clique (*44*). These algorithms usually start with a candidate clique formed by a single random vertex and then try to expand the clique size and replace some of its vertices by locally exploring the neighborhood. More precisely, the following iteration is repeated until a sufficiently good solution is found, or the maximal number of steps is reached:

1) Grow stage: Starting from a given clique, generate the set of vertices that are connected to all vertices in the clique. If this set is nonempty, select one vertex at random, possibly with large weight, and add it to the clique.

2) Swap stage: If the above set is empty, generate the set of vertices that are connected to all vertices in the clique except one (say *v*). From this new set, select a vertex at random and swap it with *v*. This gives a new clique of the same size but with different vertices, thus constituting a local change to the clique. For max weighted clique, the swapping rule also considers vertex weight.

An important aspect of the above local search is that, at each iteration step, the candidate solution is always a clique and the algorithm tries to expand it as much as possible. GBS can be included in this strategy in view of its ability to provide a starting configuration that is not a mere random vertex. A GBS output after greedy shrinking is always a clique, with a comparatively large probability of being close to the maximum clique. In case the candidate output from greedy shrinking is not the maximum clique, then it can be expanded with a few iterations of local search. Because the cliques sampled from a carefully programmed GBS device are, with high probability, larger than just a random vertex, the number of classical expansion steps is expected to be significantly reduced. This will be demonstrated with relevant numerical examples in the following section.

### Numerical results

We study the binding interaction between the tumor necrosis factor–α converting enzyme (TACE) and a thiol-containing aryl sulfonamide compound (AS). A different protein structure is studied in section S4.1. TACE was chosen because of the planar geometry of the active site cleft and its high relevance to the pharmaceutical industry. Because of its role in the release of membrane-anchored cytokines like the tumor necrosis factor–α, it is a promising drug target for the treatment of certain types of cancer, Crohn’s disease, and rheumatoid arthritis (*31*). The ligand under consideration is part of a series of thiol-containing aryl sulfonamides that exhibit potent inhibition of TACE, with a binding pose supported by a crystallographic structure (*30*). This complex provides an important testbed to benchmark our GBS-enhanced method. We use a coarse-grained representation of the TACE-AS complex that we describe below. Coarse graining is used to reduce the dimensionality of the binding interaction graph to simulate GBS with a classical computer but may be avoided when using a physical quantum device with the suitable number of modes. As we will show, our method is able to find the correct binding pose without requiring all-atom representation or simulation of the ligand/receptor complex.

The binding interaction graph for the TACE-AS complex is constructed by first extracting all the pharmacophore points on ligand and receptor using the software package rdkit (*49*). To simplify numerical simulations, we identity the relevant pairs of pharmacophore points on the ligand and receptor that are within a distance of 4 Å of each other, and whose label pairs are either hydrogen donor/acceptor, hydrophobe/hydrophobe, negative/positive charge, or aromatic/aromatic. After this procedure, we get four nodes on the ligand and six nodes on the receptor and create two labeled distance graphs as illustrated in Fig. 1. Note that the only way for two different proteins to yield the same graph is for both of them to include the same pharmacophores, all located at the same distance from each other. The knowledge-based potential is derived by combining information from PDBbind (*50*), a curated dataset of protein-ligand interactions, and the Drugscore potential (*51*). More details are presented in section S3, the resulting knowledge-based potential is shown in table S1, and the stability of the generated graphs for different values of τ is discussed in table S2.

**Fig. 4:**
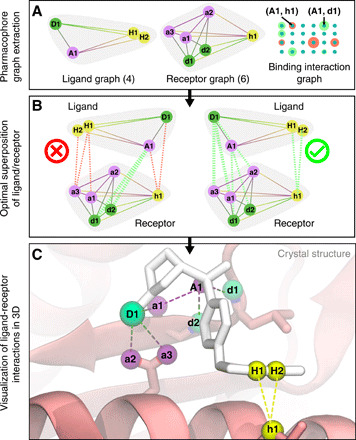
Graph-based molecular docking of an aryl sulfonamide compound to TACE. (**A**) Two labeled distance graphs—one for the aryl sulfonamide compound and one for the TACE receptor—and the resulting TACE-AS binding interaction graph. Construction of the labeled distance graph and binding interaction graph are described in Figs. 1 and 2. Pharmacophore points on the ligand and receptor are labeled with uppercase and lowercase letters, respectively. The search for the maximum vertex-weighted clique within the TACE-AS graph is illustrated in (**B**). Each clique in the TACE-AS graph corresponds to a different superposition of the ligand molecule and the TACE receptor. The correct ligand-receptor superposition corresponding to the maximum weighted clique in the TACE-AS graph is shown on the right. (**C**) Crystallographic structure of the TACE-AS complex with optimal ligand-receptor interactions correctly predicted by the maximum weighted clique. We omit the metal cofactor in the enzyme active site for visual clarity, as it was not considered as a pharmacophore point under our procedure.

Using this knowledge-based potential, we combine the two labeled distance graphs into the TACE-AS binding interaction graph, as shown in Fig. 2. A summary of our graph-based molecular docking approach is shown in Fig. 4, which includes a molecular rendering of the predicted binding interactions of the AS ligand in the TACE binding site using the crystallographic structure of this complex (Protein Data Bank: 2OI0) (*30*). These interactions correspond to the maximum vertex-weighted clique in the TACE-AS graph. This set of pharmacophore interactions can be used as constraints in a subsequent round of molecular docking to deduce three-dimensional structures of the ligand-receptor complex (*52*, *53*). We now study the search of the maximum weighted clique on the TACE-AS graph via a hierarchy of algorithms in increasing order of sophistication. As discussed previously, these are the following:

1) Random search: Generate subgraphs at random and pick the cliques with the largest weight among the outputs.

2) Greedy shrinking: Generate a large random subgraph and remove vertices until a clique is obtained. Vertices are removed by taking into account both their degree and their weight.

3) Shrinking + local search: Use the output of the greedy shrinking algorithm as the input to a DLS/PLS local search algorithm (*44*).

These form a hierarchy in the sense that random search is a subroutine of greedy shrinking, which is itself a subroutine of shrinking + local search. For each of these algorithms, we compare the performance of standard classical strategies with their quantum-classical hybrid versions introduced in the previous sections, where the random subgraph is sampled via GBS. We remark that our classical strategies are based on PLS/DLS, which are among the best-performing classical algorithms for max clique (*44*). Moreover, for a fair comparison with GBS-based approaches, the classical data are generated as follows: We first sample a subgraph size *N* from a normal distribution with the same mean ⟨*N*⟩ and variance Δ*N*^2^ as the GBS distribution, then uniformly generate a random subgraph with size *N*.

We begin our analysis with a pure GBS random search. We consider GBS with threshold detectors, which register measurement outcomes as either “no-click” (absence of photons) or “click” (presence of one or more photons). We use either a brute force approach to calculate the resulting probability distribution or, when that becomes infeasible, the exact sampling algorithm discussed in (*54*). Given the complexity of simulating GBS with classical computers, for simplicity in numerical benchmarking, we first consider the simpler case where the maximum clique size is known, so we can post-select GBS data to have a fixed number of detection clicks. This drastically simplifies numerical simulations (see section S1.4 for details) at the expense of disregarding data that would otherwise be present in an experimental setting. A similar reduction is applied to classical data for fair comparison.

**Fig. 5:**
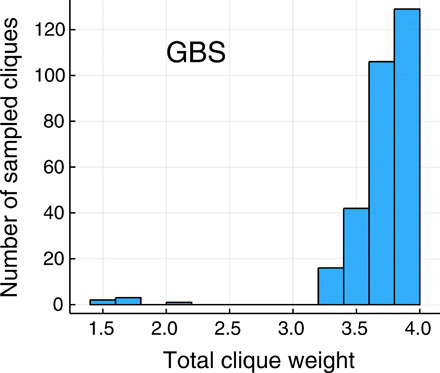
GBS random search sampling rate. Number of cliques sampled from a GBS device as a function of the total clique weight ∑_*j* ∈ *C*_*w*_*j*_. The GBS output has been post-selected to 10^5^ samples with total number of detector clicks *N* = 8. With the same number of samples (each with *N* = 8 nodes), classical random search only found three cliques (not shown), none of them with maximum weight.

For the TACE-AS binding interaction graph, the largest and heaviest cliques both have eight vertices, so we fix *N* = 8. There are a total of 19 cliques of this size in the graph (see also fig. S1 in section S4). In Fig. 5, we show the outcomes of a numerical experiment where a GBS device has been programmed to sample from the Hafnian of Ω*A*Ω, with Ω as in Eq. 3. For simplicity, we choose α = 1 in Eq. 3, although performance can be slightly improved with optimized values of α. On the other hand, the parameter *c* does not play any role in the post-selected data, but it does change the overall probability of getting samples of size *N* = 8. For comparison, we have also studied a purely classical random search, where each datum is a uniform random subgraph with *N* vertices. We observe only three cliques over 10^5^ samples. On the other hand, as shown in Fig. 5, GBS is able to produce roughly 300 cliques directly from sampling, without any classical post-processing. This indicates that the GBS distribution is favoring cliques with large weights, as intended.

Post-selecting on the number of detector clicks is an unwise strategy when using real GBS devices because it disregards otherwise useful samples. Moreover, the size of the maximum weighted clique is generally unknown. Therefore, we now use the shrinking strategy discussed in the previous sections to extract a clique from every sample, without any post-selection.

**Fig. 6:**
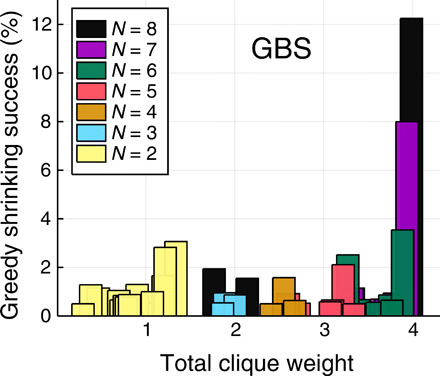
Greedy shrinking success rate. Success rate in finding cliques of different sizes (*N* = 2, …, *N*_max_), when the max clique has size *N*_max_ = 8, as a function of the total clique weight ∑_*j* ∈ *C*_*w*_*j*_. We used greedy shrinking over 10^4^ GBS samples, ignoring trivial zero-photon outcomes. Outcomes with low (<0.5%) success rate are not shown.

In Fig. 6, we study the performance of greedy shrinking with GBS data. These data consist of 10^4^ samples obtained from an exact numerical sampling algorithm (*54*). Each sample corresponds to a subgraph and, unlike Fig. 5, here, any subgraph size is considered. These results show that with GBS and greedy shrinking—a simple classical post-processing heuristic—it is possible to obtain the maximum weighted clique with sufficiently high probability. The histogram in Fig. 6 has a sharp peak corresponding to the clique of maximum size *N* = 8 and maximum weight ≈3.99. The success rate in sampling from the max weighted clique is ≈12%, and the overall sampling rate for *N* = 8 cliques is ≈19%. Greedy shrinking with purely classical random data is shown in fig. S2. Although the classical distribution is chosen to have the same mean and variance as the GBS distribution, its performance is considerably worse: The maximum weighted clique is obtained only 1% of the time compared to 12% for GBS. This shows that GBS with greedy shrinking is already able to find the maximum weight clique of the graph after only a few repetitions.

**Fig. 7:**
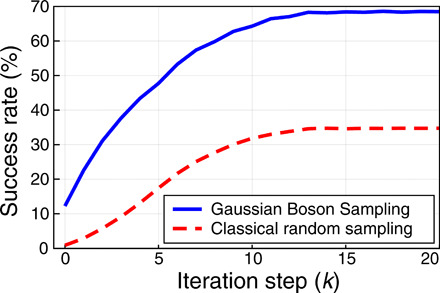
GBS versus classical success rate. Success rate in finding the maximum weighted clique after greedy shrinking and *k* expansion steps with local search. Samples are generated from either GBS or a purely classical approach. GBS maintains a significantly higher success rate over all iteration steps.

Last, we study how the cliques obtained from GBS with greedy shrinking can be enlarged or improved via local search. Figure 7 shows the performance of the hybrid GBS shrinking + local search algorithm compared to a classical strategy. The results indicate that GBS not only provides better initial estimates after greedy shrinking (zero iteration steps) but also maintains a significant margin compared to classical strategies as the number of steps is increased. After *k* = 8 local expansion steps, the probability of finding the maximum weighted clique is as high as 60%, while the classical strategy has a considerably smaller success rate of <30%. After many steps, the success rate saturates: Using GBS, the success rate gets close to 70%, while for the purely classical approach it remains under approximately 35%. The latter performance is also achieved by a “vanilla” DLS/PLS classical algorithm (*44*), where each initial configuration is a single random vertex. This shows that sampling and shrinking do not help in a fully classical strategy, whereas an advantage is observed in the quantum hybrid approach with initial GBS samples, which are expected to be closer to the real solution.

The role of noise and squeezing is discussed in section S4, where we show that GBS success rate is not diminished by the effect of noise provided that the amount of squeezing is increased accordingly. Therefore, GBS shrinking and its variant with local search are robust against noise, maintaining a significant margin compared to purely classical strategies.

## DISCUSSION

We have shown that GBS can be used to predict accurate molecular docking configurations, a central problem in pharmaceutical research and development. This is achieved by first mapping the docking problem to the task of finding large cliques in a vertex-weighted graph, then programming the GBS device to sample these cliques with high probability. This constitutes an example of the viability of near-term quantum photonic devices to tackle problems of practical interest. Further study is required to quantify the impact of improved max clique detection on the overall performance of molecular docking and to verify the applicability of this method across diverse clinically relevant ligand-receptor complexes.

Established algorithms for obtaining molecular docking configurations exist, but industrial drug discovery workflows routinely require large-scale virtual screening to enrich chemical libraries for lead candidates. In this case, a fast method for predicting docking configurations is essential. In principle, photonic devices such as Gaussian Boson Samplers can operate at very high rates and may potentially provide solutions in shorter timeframes. In addition, by sampling better random subgraphs, GBS serves as a technique to enhance the performance of classical algorithms because it increases the success rate of identifying large weighted cliques. This property is relevant and applicable in any context where identifying clusters in graphs is important beyond applications in molecular docking.

More broadly, our results establish a connection between seemingly disparate physical systems: The statistical properties of photons interacting in a linear-optical network can encode information about the spatial configuration of molecules when they combine to form larger complexes. In other words, we have found that when the interaction between fundamental particles is carefully engineered, they acquire collective properties that can be probed to perform useful tasks. A complete understanding of the capabilities of emerging quantum technologies may thus require further exploration of systems that, even if incapable of universal quantum computation, can still be programmed to exhibit properties that can be harnessed for practical applications.

## MATERIALS AND METHODS

### Gaussian Boson Sampling

Quantum systems such as the quantum harmonic oscillator or the quantized electromagnetic field can be described by phase-space methods. Here, each state is uniquely determined by a quasi-probability distribution such as the Wigner function *W*(*x*, *p*) over its position *x* and momentum *p* variables. A quantum state is called Gaussian if its Wigner function is Gaussian (*55*). Any multimode Gaussian state ρ is parametrized by its first and second moments, namely, the displacement $\alpha_{j}=\text{Tr}[\rho {\hat{\xi }}_{j}]$ and the covariance matrix σ with entries $\sigma_{\mathrm{jk}}=\text{Tr}[\{{\hat{\xi }}_{j},{\hat{\xi }}_{k}\}]/2$, where ${\hat{\xi }}_{j}$ is a vector of creation and annihilation operators: calling *M* the number of modes, ${\hat{\xi }}_{j}={\hat{a}}_{j}=({\hat{x}}_{j}+i{\hat{p}}_{j})/\sqrt{2}$ and ${\hat{\xi }}_{M+j}={\hat{a}}_{j}^{†}$ for *j* = 1, …, *M*. Gaussian quantum states are ubiquitous in quantum optics and have enabled detailed theoretical modeling and coherent manipulations in experiments (*55*).

In spite of their infinite-dimensional Hilbert space, Gaussian states can be simulated efficiently, as their evolution can be modeled by linear transformations such as Bogoliubov rotations (*56*). However, when non-Gaussian measurements are used, e.g., via photon-counting detectors (*5*, *7*) or threshold detectors (*54*), modeling measurement outcomes become extremely challenging even for supercomputers. It has been shown that, under standard complexity assumptions, sampling from the resulting probability distribution cannot be done in polynomial time using classical resources (*2*, *7*).

For a Gaussian state with zero displacement and covariance matrix σ, the GBS distribution obtained by measuring the state with photon-counting detectors is given by (*7*)

$$p(S)=\frac{\text{Haf}(A_{S})}{n_{1}!\ldots n_{M}!\sqrt{\text{det}\;(\sigma +1/2)}}$$ (4)

where $A=(\begin{array}{cc} 0 & 1\\ 1 & 0 \end{array})[1-{\left(\sigma +1/2\right)}^{-1}],$ and $A_{S}$ is a 2*N* × 2*N* submatrix of A, with $N=\sum_{j=1}^{M}n_{j}$. The set *S* = (*n*_1_, …, *n_M_*) defines a measurement outcome, where *n_j_* is the number of photons in mode *j*, and the submatrix $A_{S}$ is obtained by selecting rows and columns of A, as described in (*7*). The function Haf($A_{S}$) is the Hafnian of $A_{S}$, a matrix function that is #P-Hard to approximate for worst-case instances (*57*, *58*). For a 2*N* × 2*N* matrix *A*, it is defined as

$$\text{Haf}(A)=\sum_{ℳ\in \text{PMP}}\prod_{(i,j)\in ℳ}A_{\mathrm{ij}}$$ (5)

where PMP is the set of perfect matching permutations, namely, the possible ways of partitioning the set 1, …, 2*N* into subsets of size 2. When threshold detectors are used (*54*), the output is a binary variable *s_j_* for each mode: *s_j_* = 1 corresponds to a “click” from the *j*th detector that occurs whenever *n_j_* > 0; on the other hand, *s_j_* = 0 for *n_j_* = 0. The probability distribution with threshold detectors can be obtained by summing infinitely many probabilities from Eq. 4 or via closed-form expressions that require evaluating an exponential number of matrix determinants (*54*).

### GBS to find dense subgraphs

When *A* is the adjacency matrix of an unweighted graph *G*, the Hafnian of *A* is equal to the number of perfect matchings in *G*. Using mathematical properties of the Hafnian, it was shown in (*15*) that a GBS device can be programmed to sample from a distribution $p(S)\propto \frac{{|\text{Haf}(A_{S})|}^{2}}{c^{N}}$. The parameter *c* depends on the spectral properties of *A* and can be tuned to lower the probability of observing photon collisions, i.e., *n_j_* ≥ 2 for some *j*. More details are provided in section S1. In the collision-free subspace, *A_S_* is the adjacency matrix of the subgraph specified by the vertices *j* for which *n_j_* = 1, and Haf(*A_S_*) is equal to the number of perfect matchings in this subgraph. Therefore, a GBS device can be programmed to sample large-Hafnian subgraphs with high probability.

The density of a graph *G* is defined as the number of edges in *G* divided by the number of edges of the complete graph. Intuitively, a subgraph with a high number of perfect matchings should have a large density, a connection that was made rigorous in (*59*). This fact was used in (*13*) to show that GBS devices can be programmed to sample dense subgraphs with high probability. Hybrid quantum-classical optimization algorithms can be built by combining GBS random sampling with stochastic optimization algorithms for dense subgraph identification.

## Supplementary Material

aax1950_SM.pdf

## SUPPLEMENTARY MATERIALS

Supplementary material for this article is available at http://advances.sciencemag.org/cgi/content/full/6/23/eaax1950/DC1
