# Supplementary material for: Molecular docking with Gaussian Boson Sampling
Source: Sci Adv. 2020 Jun 5;6(23):eaax1950. doi: 10.1126/sciadv.aax1950 (PMC7274809; doi:10.1126/sciadv.aax1950)
Supplement: aax1950_SM.pdf [file aax1950_SM.pdf]

Science Advances
AAAS

advances.sciencemag.org/cgi/content/full/6/23/eaax1950/DC1

Supplementary Materials for

Molecular docking with Gaussian Boson Sampling

Leonardo Banchi*, Mark Fingerhuth, Tomas Babej, Christopher Ing, Juan Miguel Arrazola

*Corresponding author. Email: banchi.leonardo@gmail.com

Published 5 June 2020, *Sci. Adv.* **6**, eaax1950 (2020)
DOI: 10.1126/sciadv.aax1950

This PDF file includes:

Sections S1 to S4
Tables S1 and S2
Figs. S1 to S4
References

S1. TECHNICAL DETAILS

S1.1. Pure-state Gaussian Boson Sampling

Consider M single-mode squeezed states with squeezing parameter r_j injected into an $M \times M$ interferometer described by the unitary matrix U. For a pure Gaussian state, the matrix $\mathcal{A}$ entering in Eq. (4) can be decomposed as $\mathcal{A} = B \oplus B^*$, where $B = U \bigoplus_{j=1}^{M} \tanh(r_j) U^T$ [7]. Therefore, the GBS device can be programmed with any matrix B whose spectrum is contained in $[0,1]$. The adjacency matrix A of a graph normally does not have this spectral property. However, it can always be rescaled as $A = cB + d\,\mathbb{1}$ where B has the desired spectrum and c, d are suitable rescaling constants. Using properties of the Hafnian, namely $\mathrm{Haf}(A) = c^N \mathrm{Haf}(B)$ and $\mathrm{Haf}(B \oplus B^*) = |\mathrm{Haf}(B)|^2$, it was found that a GBS device can be programmed to sample from a distribution $p(S) \propto \frac{|\mathrm{Haf}(A_S)|^2}{c^N}$ [15].

S1.2. Weighted Gaussian Boson Sampling

We now introduce some technical results to show the properties of weighted Gaussian Boson Sampling.

Lemma 1. Let B be defined as in Eq. (1), where Ω is diagonal with non-zero diagonal elements $c\omega_j$ for some c. Then, B has spectrum in $[0, c]$ if $2 \max_j d_j \omega_j^2 \leq c^{-1}$.

Proof. The proof closely follows that of Lemma 1.7 in [45]. Since the Laplacian $D - A$ is positive semidefinite, so is B. Let f be any vector, then

$$\begin{aligned}\sup_f \frac{f^T B f}{f^T f} &= \sup_f \frac{f^T \Omega D^{1/2} \tilde{L} D^{1/2} \Omega f}{f^T f} = \\ &= \sup_g \frac{g^T \tilde{L} g}{g^T g} \frac{g^T g}{g^T D^{-1/2} \Omega^{-2} D^{-1/2} g} \leq \\ &\leq \sup_g \frac{2 g^T g}{g^T D^{-1/2} \Omega^{-2} D^{-1/2} g} = \\ &= \sup_f \frac{2 f^T \Omega D \Omega f}{f^T f} \leq \max_j 2 c^2 \omega_j^2 d_j \ , \end{aligned} \tag{S1}$$

where $g = D^{1/2} \Omega f$ and where we used that the spectrum of the normalized Laplacian $\tilde{L}$ is contained in $[0, 2]$. □

The main result of this section is the following decoupling theorem

Theorem 1. Let B be the matrix defined in Eq. (1). Then

$$\mathrm{Haf}(B) = \det(\Omega)\ \mathrm{Haf}(A) \ . \tag{S2}$$

Proof. The proof is based on the following expansion

$$\begin{aligned}\mathrm{Haf}(B) &\overset{(a)}{=} \sum_{M \in \mathrm{PMP}} \prod_{(ij) \in M} (B)_{ij} = \\ &\overset{(b)}{=} \sum_{M \in \mathrm{PMP}} \prod_{(ij) \in M} A_{ij} \prod_{(ij) \in M} (\omega_i \omega_j) = \\ &\overset{(c)}{=} \prod_k (\omega_k) \sum_{M \in \mathrm{PMP}} \prod_{(ij) \in M} A_{ij} \ , \end{aligned} \tag{S3}$$

where in (a) we use the definition of the Hafnian where PMP is the set of perfect matchings. Equality (b) follows from the definition of B, being the Hafnian of a matrix independent of its diagonal elements. In (b) each M contributes to the sum only if $A_{ij} \neq 0$ for all $(ij) \in M$. In the latter case the product $\prod_{(ij)\in M}(\omega_i \omega_j)$ is a product over all possible ω_j, as each vertex is visited in M only one time. In (c) we use the fact that the latter product is independent of M. □

S1.3. Biasing the number of detections

We discuss the role of parameter c in biasing the average output size. Consider a single-mode state with squeezing parameter r. Being pure, the $\mathcal{A}$ matrix is written as $\mathcal{A} = B \oplus B^*$ and, for a single mode, $B = \tanh(r)$. For maximum squeezing $r_{\max}$ we find that B can take any value in $[0, c]$ with $c = \tanh(r_{\max})$. The resulting average photon number is then $\langle N \rangle = \sinh(r)^2 = \frac{c^2}{1-c^2}$ and the variance is $\Delta N^2 \propto \langle N \rangle (1 + \langle N \rangle)$. For multiple modes the expressions are similar, though B is a matrix and $\langle N \rangle = \mathrm{Tr}[\frac{B^2}{\mathbb{1} - B^2}]$, so the normalization factor can be tuned to provide a higher rate to subgraphs of different sizes N. Although the maximum clique size is not known a priori, an estimate, e.g. based on random graphs [66], is normally enough as the large variance $\Delta N^2 \approx \langle N \rangle (1 + \langle N \rangle)$ assures that different sizes are sampled with sufficiently high rate.

Gaussian boson sampling using click detectors yields a discrete probability distribution over subsets S_N of $\{1, \ldots, M\}$ of dimension N. We write $i \in S_N$ if the ith detector "clicks" and $i \notin S_N$ otherwise. The resulting probability distribution is [54]

$$p(S_N) = \mathrm{Tr}\left[\prod_{i \in S_N} P_1^i \prod_{i \notin S_N} P_0^i\ \rho\right] \ , \tag{S4}$$

where $P_0^i = |0_i\rangle\langle 0_i|$ is the projection into the zero photon state and $P_1^i = \mathbb{1} - P_0^i$. The average number of clicks N is then

$$N = \sum_{j=1}^{M} \mathrm{Tr}[P_1^j \rho] = M - \sum_{j=1}^{M} \langle 0 | \rho_j | 0 \rangle \ , \tag{S5}$$

where ρ_j is the reduced state on mode j. Using the fidelity formula for Gaussian states [67] we then get

$$N[\sigma] = M - \sum_{j=1}^{M} \frac{1}{\sqrt{\det[\sigma_j + \mathbb{1}/2]}} , \tag{S6}$$

where σ_j is the reduced (2×2) covariance matrix for mode j. The above equation can be solved to bias the number of clicks. When the covariance matrix σ depends on the normalization factor c, we can use a simple line search algorithms to tune c such that $N[\sigma(c)]$ is equal to the desired value.

S1.4. Post-selection

Sampling from Eq. (S4) requires the calculation of all $p(S_N)$ for $N = 1, \ldots, M$. There are exponentially many of these probabilities $\mathcal{O}(2^M)$. However, if we are interested in samples of a fixed size N, then the number of $p(S_N)$ with fixed N is $\mathcal{O}(\binom{M}{N}) \approx \mathcal{O}(M^N)$. Each probability requires the evaluation of $\mathcal{O}(2^N)$ determinants, so the complexity is still exponential as a function of N [54]. However, focusing on postselection with a certain size N reduces the complexity of brute force approaches from exponential to polynomial, although the degree of this polynomial increases with N.

S1.5. Selecting parameter α

For a complete graph with $2n$ vertices the Hafnian is $h_n = \frac{2n!}{n!2^n}$. The largest Hafnian for non-complete graphs is obtained by removing an edge from the complete graph. The Hafnian is then $\frac{2n-2}{2n-1}h_n$, so this non-optimal graph is penalized by a factor $\frac{2n-2}{2n-1} \simeq 1 - \frac{1}{2n}$. A possible choice for α is to avoid a counterbalance of this term, so $1+\alpha w_{\text{tot}} < \frac{2n-1}{2n-2}$. Nonetheless, we have numerically observed that, at least for sparse graphs, the parameter α does not have to be carefully chosen, and different values of α provide the expected enhancement for the max weighted clique problem.

S2. GRAPH REPRESENTATIONS OF MOLECULAR INTERACTIONS

The binding interaction graph approach consists of two main steps, as mentioned in the main text. The first is to construct a graph that approximately models the molecular docking problem of interest by subselecting pharmacophore points in the ligand and the receptor. The second is to solve the maximum weighted clique problem for that graph. Both these steps require efficient and correct methods.

The largest weighted clique in the binding interaction graph might still seem like an abstract description of the correct binding pose. For the sake of simplicity, we will refer to the largest weighted clique as "the solution" for the rest of this section. Each node in the solution uniquely defines a contact between a pharmacophore point in the ligand and one in the receptor. Thus, three such pharmacophore points are sufficient to place the ligand into the binding site of the receptor by aligning the respective pharmacophore points within a distance smaller or equal than ϵ; one can imagine spheres of radii ϵ around each pharmacophore point in the receptor. The pharmacophore points of the ligand then need to be placed within these spheres and three pharmacophore contacts, thus, define the binding pose in real space up to an accuracy of ϵ. It follows that any additional pharmacophore contact pair (another node in the solution) will further decrease the degrees of freedom by restricting possible rotations and translations within the spheres of radii ϵ around the receptor pharmacophores.

In this section, we describe the mathematical procedure to build the binding interaction graph. We use $\mathbb{L}$ to denote the set of all labels corresponding to the individual pharmacophore point types and κ to denote the potential function $\kappa : \mathbb{L} \times \mathbb{L} \to \mathbb{R}$ that assigns an interaction strength to each pair of labels from $\mathbb{L}$.

S2.1. Labeled distance graph

Definition S2.1. *Labeled distance graph.* Let S be a set of points in three dimensional space $S = \{(x_i, y_i, z_i) \,|\, i \in I\}$ for a given index set I heuristically selecting pharmacophore points of a component (either ligand or the binding site) involved in the binding complex. Then *labeled distance graph* G_S is defined as $G_S = (V_S, E_S, \omega_S, \alpha_S)$ where

$$V_S = \{v_i \,|\, \boldsymbol{p_i} \in S\} \tag{S7}$$

is the set of vertices,

$$E_S = \{(v_i, v_j) \,|\, \boldsymbol{p_i}, \boldsymbol{p_j} \in S, i < j\} \tag{S8}$$

is the set of edges,

$$\omega_S((v_i, v_j)) = ||\, \boldsymbol{p_i} - \boldsymbol{p_j} \,|| \tag{S9}$$

is the weighting function of the edges and

$$\alpha_S : V_S \to \mathbb{L} \tag{S10}$$

is a function assigning a pharmacophore point type to each vertex.

Remark. Any labeled distance-graph is a complete graph with I vertices.

S2.2. Binding interaction graph

Let $G_L = (V_L, E_L, \omega_L, \alpha_L)$ be a ligand labeled distance graph and $G_B = (V_B, E_B, \omega_B, \alpha_B)$ labeled distance graph for the binding site. Any pair of vertices $(l_i, b_i) \in V_L \times V_B$ is then called a *contact* between G_B and G_L.

Definition S2.2. *τ flexible contact pair.* Let $c_i = (l_i, b_i)$ and $c_j = (l_j, b_j)$ be contacts between $G_L = (V_L, E_L, \omega_L, \alpha_L)$ and $G_B = (V_B, E_B, \omega_B, \alpha_B)$. Then (c_i, c_j) is a τ *flexible* contact pair between G_L and G_B if and only if $|\,\omega_L(l_i, l_j) - \omega_B(b_i, b_j)\,| \leq \tau + 2\epsilon$, where τ is the flexibility constant and ϵ is the interaction cutoff distance.

Remark. Mutual τ flexibility of contact pairs is a reflexive and symmetric relation, but not necessarily transitive.

For multiple contact pairs to be realized in the binding pose they have to not violate each other's geometric constraints and hence be pairwise τ flexible . In the following graph representation, this corresponds to a clique:

Definition S2.3. *Binding interaction graph.* Let $G_L = (V_L, E_L, \omega_L, \alpha_L)$ be a labeled distance-graph for a given ligand and $G_B = (V_B, E_B, \omega_B, \alpha_B)$ a labeled distance-graph for a given binding site. The corresponding binding interaction graph $I_{L,B}$ is defined as

$$I_{L,B} = (\mathcal{V}, \mathcal{E}, \kappa, \tau, \epsilon), \tag{S11}$$

where vertex set $\mathcal{V}$ is the set of the pairs over vertex-sets of G_L and G_B

$$\mathcal{V} = V_L \times V_B, \tag{S12}$$

and $\tau, \epsilon \in \mathbb{R}^+$ are the flexibility threshold constant and interaction cutoff distance. Then

$$\mathcal{E} \subseteq \Big\{\big((v_{l_1}, v_{b_1}), (v_{l_2}, v_{b_2})\big) \,|\, v_{l_1}, v_{l_2} \in V_L, v_{b_1}, v_{b_2} \in V_B\Big\} \tag{S13}$$

is a maximal set of τ flexible contact pairs between G_L and G_B and $\Omega : \mathcal{V} \to \mathbb{R}$ is an vertex-weighting function defined as

$$\Omega\big((v_l, v_b)\big) = \kappa\big(\alpha_L(v_l), \alpha_B(v_b)\big), \tag{S14}$$

which encodes the interaction strength between pharmacophore points corresponding to vertices v_l and v_b.

Remark. Most favourable binding pose of ligand described by labeled distance graph G_L and binding site described by labeled distance graph G_B corresponds to the heaviest vertex-weighted clique of binding interaction graph $I_{L,B}$.

S3. TACE-AS COMPLEX

The binding interaction graph used in numerical simulations is constructed as follows. First, using the software package `rdkit` [49] all the pharmacophore points on ligand and receptor are extracted. This results in 11 pharmacophore points on the AS ligand and 243 points on the TACE receptor. These sizes are potentially not too large for future GBS devices, but the classical simulation of GBS is intractable for such large problem instances [60]. Therefore, to enable numerical simulations, we subselect pharmacophore points based on the true binding pose of AS and TACE according to the following two criteria:

1. Select pairs of pharmacophore points on ligand and receptor that are within 4Å distance of each other.
2. From these pairs select the ones whose label pairs are either hydrogen donor/acceptor, hydrophobe/hydrophobe, negative/positive charge, aromatic/aromatic.

Note that in a realistic scenario the true binding pose would be unknown. However, a similar set of points could be obtained based on knowledge commonly employed in drug discovery. For example, ligand pharmacophore points can be heuristically selected and prior knowledge of the binding site location drastically reduces the number of pharmacophore points on the receptor. To reduce the number of receptor pharmacophore points even further, one could use a sliding window to study different sections of the binding site in isolation. Nevertheless, these reduction techniques will be unnecessary when physical GBS devices are built with enough modes. In the case of the TACE-AS complex, we subselect 4 points on the ligand and 6 points on the receptor and create two labelled distance graphs as illustrated in Fig. 1.

Using PDBbind [50, 61, 62], a curated dataset of protein-ligand interactions, we derive a knowledge-based pharmacophore potential. For all protein-ligand interactions, the pharmacophores on ligand and binding site are extracted with `rdkit` [49] and all pairwise distances are accumulated in a single histogram. Subsequently, the Drugscore potential [51, 63] for the six pharmacophore types (negative/positive charge, hydrogen donor/acceptor, hydrophobe and aromatic ring) is computed from the histogram as outlined in Ref. [64]. The Drugscore potential yields values in the interval $[0, 1]$ whereby favourable interactions are close to 0. Since we want to encode the correct binding pose in a maximum weighted clique, we reflect the resulting potential,

$$P_{\text{refl}}(i, j) = \max(P) - \min(P) - P_{\text{orig}}(i, j), \tag{S15}$$

such that large values in the potential encode desirable interactions. The resulting knowledge-based potential is shown in Table S1.

The final binding interaction graph for the TACE-AS complex consists of 24 nodes, so our procedure requires a GBS device with at least 24 modes. Since GBS experiments are already possible with 12 modes [65], it is reasonable to expect that devices with 24 or more modes will be reported in the near future, which would be enough to implement our TACE-AS example.

Pharmacophore type	Negative charge	Positive charge	Hydrogen-bond donor	Hydrogen-bond acceptor	Hydrophobe	Aromatic
Negative charge	0.2953					
Positive charge	0.6459	0.1596				
Hydrogen-bond donor	0.7114	0.4781	0.5244			
Hydrogen-bond acceptor	0.6450	0.7029	0.6686	0.5478		
Hydrophobe	0.1802	0.0679	0.1453	0.2317	0.0504	
Aromatic	0.0	0.1555	0.1091	0.0770	0.0795	0.1943

TABLE S1. **Knowledge-based pharmacophore potential**. Data is derived from the PDBbind dataset from 2015 [50, 61, 62]. The matrix is lower-diagonal since any potential function is symmetric.

τ	Nodes	Edges	Maximum Weighted Clique
0.7	24	154	correct
0.8	24	160	correct
0.9	24	162	correct
1.0	24	162	correct
1.1	24	162	correct
1.2	24	168	larger

TABLE S2. Stability of the binding interaction graph for different values of τ and fixed $\epsilon = 4$Å.

We conclude this section by studying the stability of the generated binding interaction graph for the TACE-AS complex for different values of the parameters. In particular, we keep $\epsilon = 4$Å fixed and vary the flexibility parameter τ. The results are shown in Table S2 where we see that for many values of τ the binding interaction graph has the same number of vertices and edges. Moreover, even when the number of edges is different, in many cases the correctness of the solution is maintained, namely the maximum weighted clique corresponds to the correct one described in the main text.

S4. SUPPLEMENTARY FIGURES

In Fig. S1 we show the position of all maximum cliques (of size $N = 8$) inside the graph, ordered from lightest to heaviest total weight. The figure shows that there are two main clusters in the graph: the top right cluster, generally with light weights, and the bottom left cluster with heavy weights. There are also a couple of intermediate cliques where these two clusters are mixed. The maximum weighted clique is shown in the bottom graph, where from node diameter we observe that it is composed by a heavy six-vertex core and two light vertices. Comparison with Fig. 5 shows that all lightweight cliques have a low occurrence rate in a carefully programmed GBS device.

In Fig. S2 we show the output of Greedy Shrinking with purely classical random data. For a fair comparison with the GBS-based approach shown in Fig. 6, the classical data are generated as follows: we first sample a subgraph size N from a normal distribution with the same mean $\langle N\rangle$ and variance ΔN^2 as the GBS distribution, then uniformly generate a random subgraph with size N. Although the resulting distribution has the same mean and variance as the GBS distribution, by comparing Fig. S2 and Fig. 6, we see that its performance is considerably worse: the maximum weighted clique is obtained only 1% of the time, compared to 12% for GBS.

In Fig. S3 we study the effect of noise and squeezing. The amount of squeezing can be controlled either by the power of a pump laser field, which induces nonlinear transformations on the squeezing mode, or by varying the detuning between the pump and cavity, which is equivalent to reducing pump power. The more intense the pump field, the larger the generated squeezing. In practice, nonlinear effects are weak and, even for large pump power, the amount of squeezing is small. Therefore, there is typically a maximum amount of achievable squeezing, which can be tuned to arbitrary values below the maximum. This does not severely limit the class of graphs that can be encoded into a GBS device, but it does restrict the average number of photons detected, which can be small if squeezing levels are low. The value $r_{\max} = 0.9702$ corresponds to an average number of detector clicks $\langle N\rangle \simeq 8$. In the lossy case, for a fair comparison, we have increased the squeezing to $r_{\max} = 0.9780$ in order to maintain the same average $\langle N\rangle \simeq 8$ and have, accordingly, samples of the same average size. As Fig. S3 shows, the success rate is not diminished by the effect of noise, provided that the amount of squeezing is increased accordingly. As a matter of fact, the noisy version with larger squeezing displays a similar success rate after greedy shrinking (iteration 0). As the iterations increase, the success rate of both noisy and noiseless GBS maintain a significant margin compared to the purely classical strategy. The slightly better performance of the noisy case is due to the larger squeezing that changes the shape of the photon distribution, while keeping comparable photon averages with the noiseless case. This analysis shows that both GBS shrinking and its variant with local search are robust against noise, maintaining a significant margin compared to purely classical strategies.

S4.1. Different protein structure

In Fig. S4 we study a different protein structure (PBD ID: 1ow7), corresponding to Paxillin LD4 motif bound

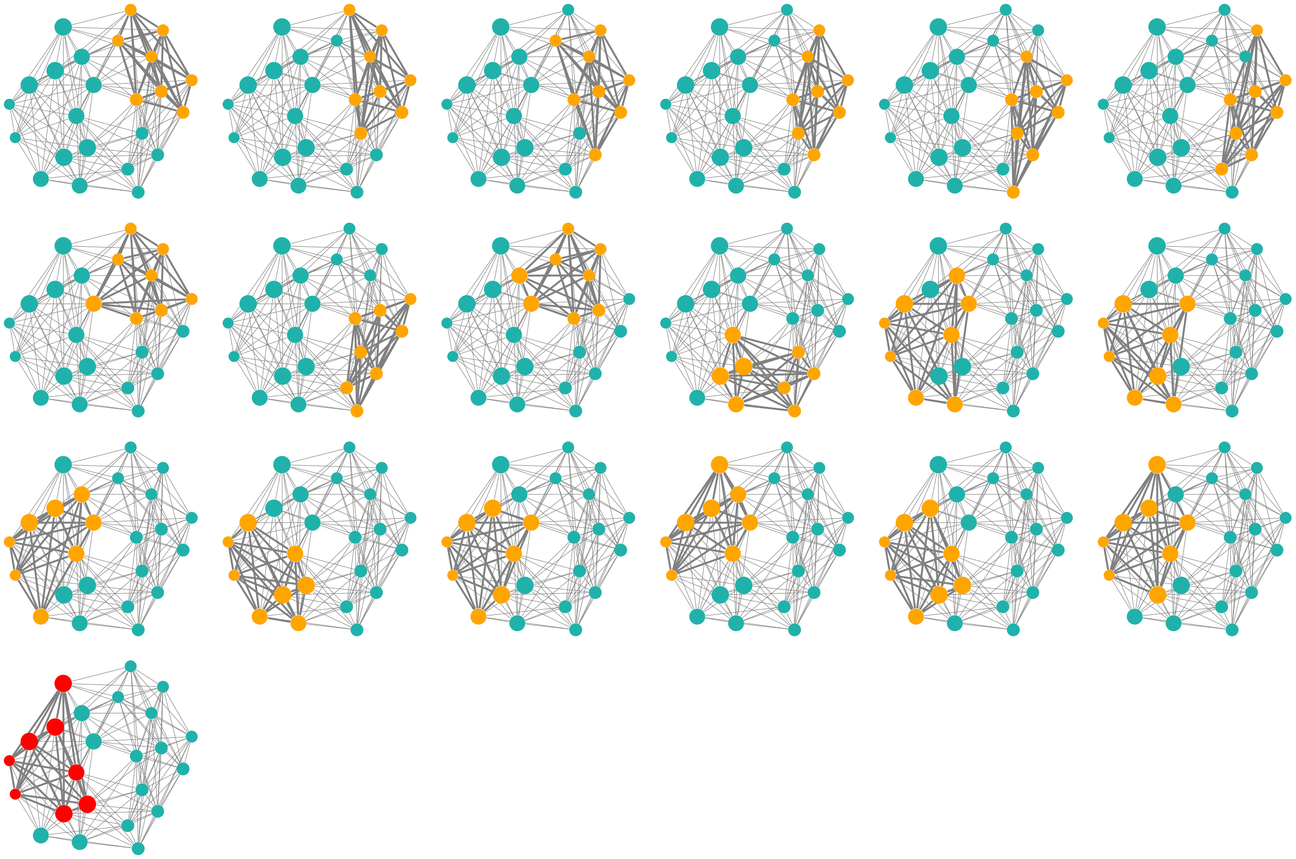

FIG. S1. **Position of all the maximum cliques.** We focus on the TACE-AS graph, where cliques are shown with orange notes, darker edges. The diameter of each vertex is proportional to its weight. The cliques are ordered from low to high total weight, starting from the top-left until the bottom-right order. The heaviest clique is shown in red in the last graph.

to the Focal Adhesion Targeting (FAT) domain of the Focal Adhesion Kinase. We run our algorithms with the same parameters used for the TACE-AS structure studied previously (PDB ID: 2oi0), and found that the resulting binding interaction graph is larger, with 30 nodes and 175 edges. The resulting maximum weighted clique, made of 6 nodes, corresponds to the optimal solution.

In Fig. S4 we study the performance of the hybrid GBS shrinking + local search algorithm, compared to a purely classical strategy. As in Fig. 7 of the main text, we observe that even without any expansion step with classical algorithms, the probability of sampling from the correct solution after greedy shrinking is negligible for purely classical strategies, while it is $\sim 6\%$ with GBS data. After a few local expansion steps, the probability of getting the right solution quickly increases for both methods, but the GBS based strategy maintains a significant margin (approximately two times larger) at any iteration step.

This analysis shows that the advantage coming from GBS based strategies is not specific to the TACE-AS complex studied in the main text, but can also be observed in other protein structures, even when the resulting binary interaction graph has a higher number of nodes.

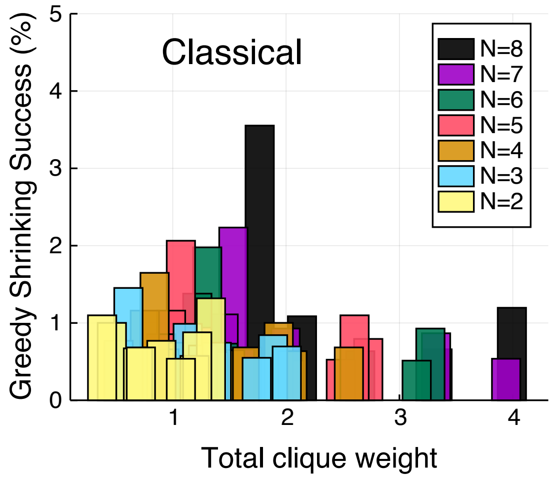

FIG. S2. **Classical greedy shrinking success rate.** Success rate in finding cliques of different sizes ($N = 2, \ldots, N_{\max}$), when the max clique has size $N_{\max} = 8$, as a function of the total clique weight $\sum_{j\in C} w_j$. We used greedy shrinking over 10^4 classical random samples. For fair comparison with GBS, classical samples where generated by first sampling a size N with same average and variance as GBS, and then selecting a random subgraph with N vertices.

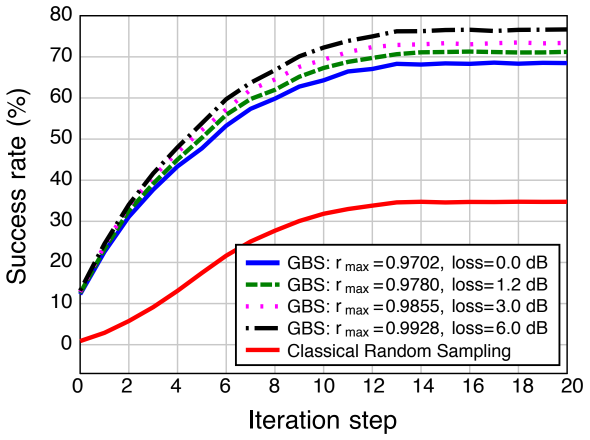

FIG. S3. **Success rate vs. squeezing and noise.** Success rate in finding the maximum weighted clique after greedy shrinking and local search. GBS is compared to a purely classical approach. For GBS, different values of squeezing and noise are considered.

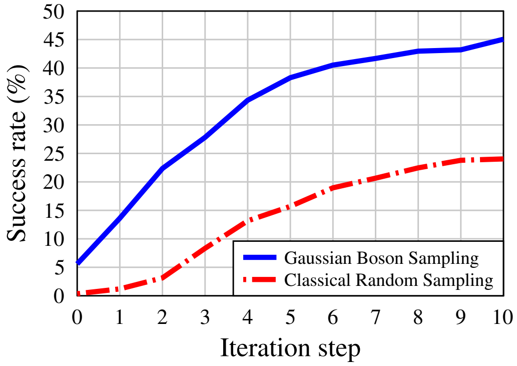

FIG. S4. **GBS vs. classical success rate for "1ow7".** Success rate in finding the maximum weighted clique after greedy shrinking and different expansion steps with local search, as in Fig. 7.

REFERENCES AND NOTES

1. R. P. Feynman, Simulating physics with computers. *Int. J. Theor. Phys.* **21**, 467–488 (1982).

2. S. Aaronson, A. Arkhipov, The computational complexity of linear optics, in *Proceedings of The Forty-Third Annual ACM Symposium on Theory of Computing* (ACM, 2011), pp. 333–342.

3. P. Clifford, R. Clifford, The classical complexity of boson sampling, in *Proceedings of the Twenty-Ninth Annual ACM-SIAM Symposium on Discrete Algorithms* (Society for Industrial and Applied Mathematics, 2018), pp. 146–155.

4. A. Neville, C. Sparrow, R. Clifford, E. Johnston, P. M. Birchall, A. Montanaro, A. Laing, Classical boson sampling algorithms with superior performance to near-term experiments. *Nat. Phys.* **13**, 1153–1157 (2017).

5. A. P. Lund, A. Laing, S. Rahimi-Keshari, T. Rudolph, J. L. O'Brien, T. C. Ralph, Boson sampling from a Gaussian state. *Phys. Rev. Lett.* **113**, 100502 (2014).

6. M. Bentivegna, N. Spagnolo, C. Vitelli, F. Flamini, N. Viggianiello, L. Latmiral, P. Mataloni, D. J. Brod, E. F. Galvão, A. Crespi, R. Ramponi, R. Osellame, F. Sciarrino, Experimental scattershot Boson sampling. *Sci. Adv.* **1**, e1400255 (2015).

7. C. S. Hamilton, R. Kruse, L. Sansoni, S. Barkhofen, C. Silberhorn, I. Jex, Gaussian Boson sampling. *Phys. Rev. Lett.* **119**, 170501 (2017).

8. Z. Vernon, N. Quesada, M. Liscidini, B. Morrison, M. Menotti, K. Tan, J. E. Sipe, Scalable squeezed light source for continuous variable quantum sampling. *Phys. Rev. Appl.* **12**, 064024 (2018).

9. S. Wolfram, Undecidability and intractability in theoretical physics. *Phys. Rev. Lett.* **54**, 735–738 (1985).

10. J. Huh, G. G. Guerreschi, B. Peropadre, J. R. McClean, A. Aspuru-Guzik, Boson sampling for molecular vibronic spectra. *Nat. Photon.* **9**, 615–620 (2015).

11. W. R. Clements, J. J. Renema, A Eckstein, A. A. Valido, A. Lita, T. Gerrits, S. W. Nam, W. S. Kolthammer, J. Huh, I. A. Walmsley, Approximating vibronic spectroscopy with imperfect quantum optics. *J. Phys. B At. Mol. Phys.* **51**, 245503 (2017).

12. C. Sparrow, E. Martín-López, N. Maraviglia, A. Neville, C. Harrold, J. Carolan, Y. N. Joglekar, T. Hashimoto, N. Matsuda, J. L. O'Brien, D. P. Tew, A. Laing, Simulating the vibrational quantum dynamics of molecules using photonics. *Nature* **557**, 660–667 (2018).

13. J. M. Arrazola, T. R. Bromley, Using Gaussian Boson sampling to find dense subgraphs.

Phys. Rev. Lett. **121**, 030503 (2018).

14. J. M. Arrazola, T. R. Bromley, P. Rebentrost, Quantum approximate optimization with Gaussian Boson sampling. *Phys. Rev. A* **98**, 012322 (2018).

15. K. Brádler, P.-L. Dallaire-Demers, P. Rebentrost, D. Su, C. Weedbrook, Gaussian boson sampling for perfect matchings of arbitrary graphs. *Phys. Rev. A* **98**, 032310 (2018).

16. D. B. Kitchen, H. Decornez, J. R. Furr, J. Bajorath, Docking and scoring in virtual screening for drug discovery: Methods and applications. *Nat. Rev. Drug Discov.* **3**, 935–949 (2004).

17. X.-Y. Meng, H.-X. Zhang, M. Mezei, M. Cui, Molecular docking: A powerful approach for structure-based drug discovery. *Curr. Comput. Aided Drug Des.* **7**, 146–157 (2011).

18. R. L. DesJarlais, R. P. Sheridan, J. S. Dixon, I. D. Kuntz, R. Venkataraghavan, Docking flexible ligands to macromolecular receptors by molecular shape. *J. Med. Chem.* **29**, 2149–2153 (1986).

19. B. K. Shoichet, I. D. Kuntz, D. L. Bodian, Molecular docking using shape descriptors. *J. Comput. Chem.* **13**, 380–397 (1992).

20. B. K. Shoichet, I. D. Kuntz, Matching chemistry and shape in molecular docking. *Protein Eng.* **6**, 723–732 (1993).

21. R. Dias, W. F. J. de Azevedo, Molecular docking algorithms. *Curr. Drug Targets* **9**, 1040–1047 (2008).

22. H. Alonso, A. A. Bliznyuk, J. E. Gready, Combining docking and molecular dynamic simulations in drug design. *Med. Res. Rev.* **26**, 531–568 (2006).

23. B. K. Shoichet, Virtual screening of chemical libraries. *Nature* **432**, 862–865 (2004).

24. M. Hernandez, M. Aramon, Enhancing quantum annealing performance for the molecular similarity problem. *Quant. Inf. Proc.* **16**, 133 (2017).

25. M. Hernandez, G. L. Gan, K. Linvill, C. Dukatz, J. Feng, G. Bhisetti, A quantum-inspired method for three-dimensional ligand-based virtual screening. *J. Chem. Inf. Model.* **59**, 4475–4485 (2019).

26. F. S. Kuhl, G. M. Crippen, D. K. Friesen, A combinatorial algorithm for calculating ligand binding. *J. Comput. Chem.* **5**, 24–34 (1984).

27. I. D. Kuntz, J. M. Blaney, S. J. Oatley, R. Langridge, T. E. Ferrin, A geometric approach to macromolecule-ligand interactions. *J. Mol. Biol.* **161**, 269–288 (1982).

28. I. Halperin, B. Ma, H. Wolfson, R. Nussinov, Principles of docking: An overview of search

algorithms and a guide to scoring functions. *Proteins* **47**, 409–443 (2002).

29. G. L. Warren, C. W. Andrews, A.-M. Capelli, B. Clarke, J. L. Londe, M. H. Lambert, M. Lindvall, N. Nevins, S. F. Semus, S. Senger, G. Tedesco, I. D. Wall, J. M. Woolven, C. E. Peishoff, M. S. Head, A critical assessment of docking programs and scoring functions. *J. Med. Chem.* **49**, 5912–5931 (2006).
30. B. G. Rao, U. K. Bandarage, T. Wang, J. H. Come, E. Perola, Y. Wei, S.-K. Tian, J. O. Saunders, Novel thiol-based TACE inhibitors: Rational design, synthesis, and SAR of thiol-containing aryl sulfonamides. *Bioorg. Med. Chem. Lett.* **17**, 2250–2253 (2007).
31. M. L. Moss, L. Sklair-Tavron, R. Nudelman, Drug Insight: Tumor necrosis factor-converting enzyme as a pharmaceutical target for rheumatoid arthritis. *Nat. Clin. Pract. Rheumatol.* **4**, 300–309 (2008).
32. S. Kalyaanamoorthy, Y.-P. P. Chen, Structure-based drug design to augment hit discovery. *Drug Discov. Today* **16**, 831–839 (2011).
33. S.-Y. Yue, Distance-constrained molecular docking by simulated annealing. *Protein Eng.* **4**, 177–184 (1990).
34. O. Trott, A. J. Olson, AutoDock Vina: Improving the speed and accuracy of docking with a new scoring function, efficient optimization, and multithreading. *J. Comput. Chem.* **31**, 455–461 (2009).
35. M. Rarey, B. Kramer, T. Lengauer, G. Klebe, A fast flexible docking method using an incremental construction algorithm. *J. Mol. Biol.* **261**, 470–489 (1996).
36. M. D. Miller, S. K. Kearsley, D. J. Underwood, R. P. Sheridan, FLOG: A system to select 'quasi-flexible' ligands complementary to a receptor of known three-dimensional structure. *J. Comput. Aided Mol. Des.* **8**, 153–174 (1994).
37. T. J. A. Ewing, I. D. Kuntz, Critical evaluation of search algorithms for automated molecular docking and database screening. *J. Comput. Chem.* **18**, 1175–1189 (1997).
38. M. D. Miller, R. P. Sheridan, S. K. Kearsley, SQ: A program for rapidly producing pharmacophorically relevent molecular superpositions. *J. Med. Chem.* **42**, 1505–1514 (1999).
39. S.-Y. Yang, Pharmacophore modeling and applications in drug discovery: Challenges and recent advances. *Drug Discov. Today* **15**, 444–450 (2010).
40. A. M. Poole, R. Ranganathan, Knowledge-based potentials in protein design. *Curr. Opin. Struct. Biol.* **16**, 508–513 (2006).
41. J. Mintseris, B. Pierce, K. Wiehe, R. Anderson, R. Chen, Z. Weng, Integrating statistical pair

potentials into protein complex prediction. *Proteins* **69**, 511–520 (2007).

42. H. Gohlke, G. Klebe, Statistical potentials and scoring functions applied to protein–ligand binding. *Curr. Opin. Struct. Biol.* **11**, 231–235 (2001).
43. R. M. Karp, Reducibility among Combinatorial Problems, in *Complexity of Computer Computations* (Springer, 1972), pp. 85–103.
44. Q. Wu, J.-K. Hao, A review on algorithms for maximum clique problems. *Eur. J. Oper. Res.* **242**, 693–709 (2015).
45. F. R. Chung, F. C. Graham, *Spectral Graph Theory* (American Mathematical Society, 1997).
46. I. Gutman, M. Ghorbani, Some properties of the Narumi–Katayama index. *Appl. Math. Lett.* **25**, 1435–1438 (2012).
47. W. Pullan, H. H. Hoos, Dynamic local search for the maximum clique problem. *J. Artif. Intell. Res.* **25**, 159–185 (2006).
48. W. Pullan, Phased local search for the maximum clique problem. *J. Comb. Optim.* **12**, 303–323 (2006).
49. G. Landrum, RDKit: Cheminformatics and machine learning software (2006). RDKit: Open-Source Cheminformatics Software; http://rdkit.org/.
50. R. Wang, X. Fang, Y. Lu, S. Wang, The PDBbind database: Collection of binding affinities for protein−ligand complexes with known three-dimensional structures. *J. Med. Chem.* **47**, 2977–2980 (2004).
51. H. Gohlke, M. Hendlich, G. Klebe, Knowledge-based scoring function to predict protein-ligand interactions. *J. Mol. Biol.* **295**, 337–356 (2000).
52. S. A. Hindle, M. Rarey, C. Buning, T. Lengauer, Flexible docking under pharmacophore type constraints. *J. Comput. Aided Mol. Des.* **16**, 129–149 (2002).
53. M. L. Verdonk, V. Berdini, M. J. Hartshorn, W. T. M. Mooij, C. W. Murray, R. D. Taylor, P. Watson, Virtual screening using protein-ligand docking: Avoiding artificial enrichment. *J. Chem. Inf. Comput. Sci.* **44**, 793–806 (2004).
54. N. Quesada, J. M. Arrazola, N. Killoran, Gaussian Boson sampling using threshold detectors. *Phys. Rev. A* **98**, 062322 (2018).
55. C. Weedbrook, S. Pirandola, R. García-Patrón, N. J. Cerf, T. C. Ralph, J. H. Shapiro, S. Lloyd, Gaussian quantum information. *Rev. Mod. Phys.* **84**, 621–669 (2012).
56. S. D. Bartlett, B. C. Sanders, S. L. Braunstein, K. Nemoto, Efficient classical simulation of

continuous variable quantum information processes. *Phys. Rev. Lett.* **88**, 097904 (2002).

57. E. R. Caianiello, On quantum field theory—I: Explicit solution of Dyson’s equation in electrodynamics without use of feynman graphs. *Il Nuovo Cimento (1943-1954)* **10**, 1634–1652 (1953).

58. A. Björklund, B. Gupt, N. Quesada, A faster hafnian formula for complex matrices and its benchmarking on a supercomputer. *J. Exp. Algorithmics* **24**, 1 (2018).

59. M. Aaghabali, S. Akbari, S. Friedland, K. Markström, Z. Tajfirouz, Upper bounds on the number of perfect matchings and directed 2-factors in graphs with given number of vertices and edges. *Eur. J. Combinatorics* **45**, 132–144 (2015).

60. B. Gupt, J. M. Arrazola, N. Quesada, T. R. Bromley, Classical benchmarking of Gaussian Boson Sampling on the Titan supercomputer. arXiv:1810.00900 [quant-ph] (1 October 2018).

61. Z. Liu, Y. Li, L. Han, J. Li, J. Liu, Z. Zhao, W. Nie, Y. Liu, R. Wang, PDB-wide collection of binding data: Current status of the PDBbind database. *Bioinformatics* **31**, 405–412 (2014).

62. R. Wang, X. Fang, Y. Lu, C.-Y. Yang, S. Wang, The PDBbind database: Methodologies and updates. *J. Med. Chem.* **48**, 4111–4119 (2005).

63. H. Gohlke, M. Hendlich, G. Klebe, Predicting binding modes, binding affinities and `hot spots' for protein-ligand complexes using a knowledge-based scoring function. *Perspect. Drug Discov. Design* **20**, 115–144 (2000).

64. W. T. M. Mooij, M. L. Verdonk, General and targeted statistical potentials for protein-ligand interactions. *Proteins* **61**, 272–287 (2005).

65. H.-S. Zhong, L.-C. Peng, Y. Li, Y. Hu, W. Li, J. Qin, D. Wu, W. Zhang, H. Li, L. Zhang, Z. Wang, L. You, X. Jiang, L. Li, N.-L. Liu, J. P. Dowling, C.-Y. Lu, J.-W. Pan, Experimental Gaussian Boson sampling. *Sci. Bull.* **64**, 511–515 (2019).

66. B. Bollobás, P. Erdös, Cliques in random graphs, in *Mathematical Proceedings of the Cambridge Philosophical Society* (Cambridge Univ. Press, 1976), vol. 80, pp. 419–427.

67. L. Banchi, S. L. Braunstein, S. Pirandola, Quantum fidelity for arbitrary Gaussian States. *Phys. Rev. Lett.* **115**, 260501 (2015).
